# Measuring the One Health impacts associated with creating access to veterinary care before and during the COVID-19 pandemic

**DOI:** 10.3389/fpubh.2024.1454866

**Published:** 2024-12-09

**Authors:** Sloane M. Hawes, Kaleigh M. O’Reilly, Tess M. Mascitelli, Jordan Winczewski, Romi Dazzio, Amanda Arrington, Kevin N. Morris

**Affiliations:** ^1^Institute for Human-Animal Connection, Graduate School of Social Work, University of Denver, Denver, CO, United States; ^2^Pets for Life, The Humane Society of the United States, Washington, DC, United States

**Keywords:** One Health, social determinants of health, social determinants of animal health, companion animals, COVID-19, access to veterinary care, One Health Community Assessment

## Abstract

Integrating community perceptions into One Health assessments is critical to understanding the structural barriers that create disproportionate health outcomes for community members, their pets, and the ecosystems that encompass them, particularly in historically marginalized and under-resourced communities. The validated One Health Community Assessment (OHCA) survey instrument was used to evaluate the associated impacts of The Humane Society of the United States’ Pets for Life (PFL) programming on communities’ perceptions of One Health. This evaluation took place across two phases, totaling four years. In phase one (May 2018 – December 2019), the PFL intervention was administered to one urban and one rural under-resourced community, while two demographically-paired communities served as comparison sites. Five OHCA subscales (human health, pet health, environmental health, community health, perceived links) were employed to measure changes in perceptions of One Health and fourteen OHCA items were used to measure perceptions of access to human healthcare, pet care, and the environment. Initiation of the confirmatory second phase of the study (May 2020–October 2021), in which all four communities received the intervention, coincided with the onset of the COVID-19 pandemic. The pandemic and its resulting public health mandates hindered both PFL programming and data collection. Generalized Estimating Equations were employed in both the first and second phase analyses to model changes in perceptions of One Health associated with the PFL intervention. In the study’s first phase, PFL in the urban community was associated with significant increases in perceptions of community health and environmental health, and perceived access to human health care, pet care, and the environment. The presence of PFL during the study’s second phase was associated with increased perceptions of environmental health. The variables of PFL and the pandemic were not able to be isolated within the analyses. However, due to the severe, negative One Health implications associated with the COVID-19 pandemic, the phase two results were interpreted from the perspective of the pandemic being the largest driver of the results. The results are consistent with previous research on the effects of the pandemic on community perceptions of health. These findings offer initial support for the hypothesis that deployment of resources focused on companion animals may affect perceptions across the One Health triad and confirms previous research on effects of the COVID-19 pandemic.

## 1 Introduction

The One Health framework states that the health of humans, other animals (hereafter referred to as animals), and the environment they share are interconnected, and has been central to guiding holistic approaches to supporting ecosystem health throughout the twenty-first century (1–3). Currently, zoonosis, comparative medicine, and food safety are the primary foci of One Health initiatives, with many of these efforts positively impacting health outcomes on the global scale (4). However, two limitations that consistently surface are a lack of systems-level, or holistic, analysis of the effects of the human-animal bond (e.g., pet ownership) and an overwhelmingly anthropocentric focus of One Health programs. Siloed approaches prevent the realization of holistic interventions to interconnected health issues across socioecological systems, creating vulnerabilities within the framework (5–7). Several scholars have also called for more nuanced One Health initiatives and research that consider the cultural, social, political, and material contexts in which health interventions for humans, animals, and the environment take place (8). This is particularly relevant regarding historically marginalized and under-resourced communities that are most affected by One Health challenges (9). A few studies have centrally integrated community priorities, knowledge, and practices into the local-level application of One Health interventions (8–11). Several of these studies found that community-centric strategies working toward adaptation and resilience were more effective when those with on-the-ground experience were integrated into the process of gathering and synthesizing data.

Crucial to successfully integrating community priorities into One Health programming and research is assessing residents’ perceptions regarding structural barriers to accessing each domain of the triad. This concept is exemplified within human healthcare settings where understanding individuals’ perspectives is an integral factor in program evaluation (12). Individual-level attitudes toward healthcare resources can identify the need for specific community supports that facilitate transitioning from not just “having” access but to “gaining” access (13, 14). However, analyses of community perspectives regarding barriers to health services are complex because they can vary significantly from person to person due to the context of their specific health needs and the physical and social environments in which they live (13). These structural barriers understood as the social determinants of health (SDH), consists of one’s community and social interactions, economic stability, neighborhood and the built environment, and the quality and accessibility of education and health care. According to the Centers for Disease Control and Prevention, SDH contribute to a wide range of health disparities and inequities among human populations, particularly in under-resourced communities (15).

Similar to SDH for humans, social determinants of animal health (SDAH) describe inequities in veterinary health, among companion animals (also referred to as pets) specifically, that extend beyond concerns of welfare traditionally outlined in the “Five Freedoms” (e.g., humane management, care, handling) (16–18). Like health outcomes in human populations, the occurrence of animal disease and an owner’s usage of animal care services greatly depends upon the circumstances in which animals “are born, grow, live, work, and age” (16, 19). Therefore, SDH and SDAH collectively outline the social factors, barriers, and intervention points that people and their pets encounter as they try to gain access to health care. Understanding the direct and indirect relationships these social determinants have on attaining optimal health for humans, animals, and the environment could create more holistic One Health interventions.

Integrating a community’s lived experience and perception of SDHs and SDAHs into One Health analyses could better illuminate how systemic imbalances in power, resource distribution, and exposure to health hazards are creating disproportionate health outcomes for human communities, their pets, and the ecologies in which they are embedded (16). A tool for such analyses has been created through *The development and validation of the One Health Community Assessment* (OHCA) (20). This quantitative instrument was designed using qualitative interviews with residents in an under-resourced community to capture perspectives of individual domains and interconnections within the One Health framework. The OHCA was further refined and validated using the data collected across the four-years of this study (20). The instrument includes measurement across five subscales of human health, pet health, environmental health, community health, and the perceived links between the domains of One Health. Groups of items embedded within these subscales assess perceptions of access to veterinary care, human health care, and the environment (21). Pre-study validation of the resulting instrument was initiated using pilot data and then confirmed using data from the study reported here (20). The OHCA offers a unique method to measure attitudes across a breadth of preventive health interventions, including pet ownership. The OHCA works by capturing how, SDH, and SDAH impact an individual’s health-promoting behaviors for themselves and their animals, including perceived barriers to accessing health care.

The Humane Society of the United States’ Pets for Life (PFL) program is one of the longest-running and highly protocolized initiatives to improve access to pet support services in under-resourced communities in the U.S. Through partnerships with local animal welfare service organizations, PFL offers no-cost or heavily subsidized veterinary care and pet supplies (e.g., food, leashes, outdoor housing), transportation to and from appointments, and bilingual staff members to work with the community to build and maintain relationships with pet owners. PFL programming has been sustainably implemented in communities through a variety of funding mechanisms to ensure long-term service provision. With this foundation, since 2011, PFL has served over 60 communities and 315,000 pets by providing over 1.175 million veterinary services, supplies, and medications across in the U.S. and Canada (22). The PFL model offers an opportunity to study how the intentional deployment of substantial resources toward pet well-being impacts a community’s perceptions across the One Health triad.

The study addresses several limitations in contemporary One Health research by focusing on resource allocation to the animal, as opposed to human, domain of the triad and measuring the effects across systems, particularly in under-resourced communities. In the present study, the OHCA instrument (20) is used to assess how the presence of PFL in under-resourced communities impacted individuals’ overall perceptions of One Health and their perceptions of access to three components of the One Health triad. The overarching goal is to better understand how PFL is impacting communities’ perspectives across a variety of health measures and to use that information to optimize service access and intended impacts. Within the first two-year phase of the four-year study design, we hypothesized that community members in under-resourced communities receiving the PFL intervention would have more positive perceptions of both the individual One Health subscales and of access to human and environmental health resources compared to those in a similar community not receiving PFL services. Hawes et al. (21) previously reported on the impact of PFL on perceptions of access to veterinary care and pet-supportive services, finding a positive change in perceptions in the urban community receiving the intervention. Findings from this study (21) will be used as comparators to the measured perceptions of access to human health care and the environment in this study. In the second two-year phase, when the original comparison communities also began receiving PFL services, we hypothesized that all the communities would have elevated perceptions of the five different OHCA subscales associated with the PFL intervention across the four-year study. However, the COVID-19 outbreak impacted this confirmatory period of the study, essentially creating before and during pandemic data collection periods of equivalent duration. Although not its original intent, the reported study likely measured the potential effects of focused resource deployment into the animal component of the One Health triad during the first half of data collection and the effects of a major zoonotic pandemic on community perceptions of One Health during the second half.

## 2 Materials and methods

### 2.1 Study sites

Four communities were selected to participate in the four-year study as previously described in Hawes et al. (21). Two urban communities, Madison, WI (53713) and Seattle, WA (98108), and two rural communities, Granger, WA (98932) and Wilder, ID (83676), were chosen for the study based on intra-pair similarities in the demographic selection criteria (21). Each of the study sites had higher numbers of individuals living below the federal poverty line and higher racial and ethnic diversity than the U.S. average (23). Within this study, an urban community was defined as a populated area metropolitan area with substantial residential, commercial, and transportation infrastructure. A rural community was defined as an agricultural area with low population density (24). In the first year of this study, a detailed assessment of pet ownership was conducted to understand how many households may benefit from the PFL intervention (25). The measured pet ownership rate for each community was: Madison 58.6%, Seattle 48.1%, Granger 64.7%, and Wilder 64.9%.

The study was conducted over a four-year period (2018–2021) under a University of Denver IRB-approved consent and data collection protocol (DU IRB Protocol 1234950). In the first phase of the study (2018–2019), one urban and one rural site, Madison, WI and Granger, WA, were selected to receive the PFL program while the other sites, Seattle, WA and Wilder, ID, served as comparison communities. In the second phase of the study (2020–2021), Madison, WI and Granger, WA continued to receive the PFL program, and Seattle, WA and Wilder, ID began receiving the intervention. Thus, in the second phase of the study, the pre-intervention surveys served as the comparison.

### 2.2 Intervention

The intervention in this study, PFL, generates support services for people and their pets in under-resourced communities using a consistent program model that includes conducting strategic door-to-door outreach, building a consistent presence, and utilizing a comprehensive follow-up process. PFL provides services that include, but are not limited to, veterinary wellness and sick/urgent care, spay/neuter, pet food, pet supplies, transportation to and from services, and information. All services provided are heavily subsidized or no cost to clients residing in the focus community. Depth and breadth of resources offered through the program vary by community based on the capacity of the operating organization, service partner providers, and community feedback. When PFL adds a new mentorship partner, the first year of the program implementation is critical in preparing the organization to operate a community-based effort through establishing strong community relationships. The first year is comprised of the organization attending a two-day intensive training at an existing program location and the national PFL team then visiting the new local community. This first year also includes identifying an initial focus area within the community, creating an outreach schedule, and developing a cadence to service provision, all of which rely on face-to-face interactions with people and their pets. The intervention initiation and ongoing service provision in the initial two study sites (Madison, WI and Granger, WA) were consistent with this standard PFL programming during the first two years of the study.

With the onset of the COVID-19 pandemic in March 2020, PFL had to shift its approach dramatically to both program initiation and service provision nationwide. The organizations partnering with PFL during the height of the pandemic experienced a long list of challenges and, for a period, complete uncertainty regarding ongoing program capacity. Legally mandated shelter-in-place orders disallowed PFL travel and outreach, and service providers, such as veterinary clinics, suspended what was determined as non-essential operations. The PFL program had to make significant shifts in operations to ensure the safety of staff and the community while maintaining community connections and ensuring timely delivery of the most critical resources people needed for their pets. During lockdown, PFL stayed in touch with community clients via phone and text, made no-contact deliveries of pet food and supplies, and utilized veterinary telemedicine where allowed. Trying to build and maintain relationships in lieu of PFL’s traditional face-to-face outreach methods was challenging. This particularly impacted the two second phase study sites (Seattle, WA and Wilder, ID) as they tried to build new community relationships starting in 2020. Moreover, when outreach could resume, wearing masks, social distancing, and a general fear for one’s health, made authentic relationships and service utilization within the typical PFL model difficult. Veterinary care and other services were also extremely limited, resulting in long wait times for appointments, surgeries, and treatments. Therefore, due to the COVID-19 pandemic, the PFL intervention was not implemented in a consistent manner across the four-year study period in the study sites with respect to rates of initiation, magnitude, and timely service provision. Important to interpreting the finding from the study, these pandemic-related impacts bisected the four year study period.

### 2.3 Instrumentation

The OHCA instrument was developed prior to the study using an exploratory sequential mixed methods approach. A series of 115 items were generated from themes coded from transcripts of open-ended interviews with residents in the PFL site in Denver, CO that explored One Health concepts relevant to their community. These items were initially validated using an exploratory factor analysis of 105 pilot surveys prior to initiation of data collection in this study (20). A Spanish translation was also developed and used in the reported study (26). Each survey item was structured to allow rating on a 5-point Likert scale, with 1 = “Strongly Disagree,” 2 = “Disagree,” 3 = “Neutral,” 4 = “Agree,” and 5 = “Strongly Agree,” with additional response options for “Prefer not to answer” and “Not applicable.”

The 115 OHCA items are grouped into five subscales: human health, pet health, environmental health, community health, and the perceived links between the domains of One Health (20). The human health subscale contained 22 items about perceptions regarding accessibility, affordability, satisfaction with and use of healthcare services, exercise frequency, progress toward personal goals, and levels of worry regarding their health. The pet health subscale contained 25 items about perceptions regarding accessibility, affordability, satisfaction with, and use of pet care services, their pet’s vaccination and sterilization status, their pet’s exercise frequency, and levels of worry regarding their pet’s health. The environmental health subscale contained 15 items about perceptions of the accessibility and affordability of options to enjoy nature, the presence of plants and wildlife, respect for the environment, and the practice of recycling, composting, and disposal of hazardous materials. The community health subscale contained 13 items about perceptions of neighborhood or community cohesion, trust in the government, and safety. The perceived links subscale contained 20 items about perceptions of relationships with and impact on one’s pets, the environment, and vice versa. Sets of items were used to assess perceived access to human health care (six items), pet health care (six items), and the environment (two items). Psychometric analysis of the first two years of data from the reported study found high reliability, with Cronbach’s as of 0.842, 0.899, 0.789, 0.897, 0.762 for the human health, pet health, environmental health, community health, and perceived links subscales, respectively (20).

### 2.4 Data collection

CBRAs were hired at each of the four sites and employed with study-specific grant funds through the areas’ local animal sheltering organization implementing PFL (Dane County Humane Society for Madison, WI; Seattle Humane for Seattle, WA; Yakima Humane Society for Granger, WA; and Idaho Humane Society for Wilder, ID). These CBRAs were living in or near their focus community, had previous research experience, and exhibited strong skills in building rapport with diverse populations. CBRAs received intensive training on culturally appropriate research methods from the project team. The project manager conducted regular fidelity checks throughout the study period to ensure CBRAs were complying with the research protocol.

CBRAs recruited study participants through door-to-door outreach using systematic sampling grids that included half of the households in the urban communities and all households in the rural communities. Three attempts of contact were made at each household in the sampling grid, with each attempt made on different days of the week and times of day to accommodate community members’ schedules and maximize contact rates. When a CBRA made contact at a household, they described the purpose of the study, evaluated if the participant met the study inclusion criteria, and recruited participation by offering a $20 VISA gift card. Eligibility criteria included: living in a household within one of the four focus community’s zip codes (53713, 98108, 98932, or 83676) and current pet ownership or pet ownership within the past 12 months. For the purposes of this study, pet ownership was defined as any common household pet (e.g., dog, cat) and excluded livestock or farm animals (e.g., horses, chickens, cattle). If individuals met the inclusion criteria and consented to participate in the study, the CBRA first collected human demographic data, including preferred language, sex, age, ethnicity, highest level of education, household income, born in the U.S. and pet demographic data, including pet(s) names, type and breed of pet(s), and pet(s) source of acquisition. The CBRA then orally administered the OHCA in the participant’s preferred language of English or Spanish. Responses were recorded on an electronic tablet and transferred to a secure data management system (REDCap) hosted at the University of Denver (27). Participants could complete one survey per year for each of the four years of the study as long as they continued to meet the eligibility criteria. Therefore, if they resided in the initial intervention communities (Madison, WI and Granger, WA) they could complete up to four surveys associated with the presence of PFL, whereas if they resided in the initial comparison communities (Seattle, WA and Wilder, ID) they could complete up to two surveys not associated with the PFL intervention and up to two surveys associated with the PFL intervention.

During the first phase of the study, OHCA surveys were administered in person across all four study sites. The first-year collection took place from May 2018 to December 2018, and the second-year collection took place from May 2019 to April 2020. At the onset of the COVID-19 pandemic, CBRAs had to shift data collection, under a specific IRB-approved COVID-19 protocol, to comply with governmental public health mandates. While existing participants were maintained and data was still able to be collected in person during 2020 and 2021 (year three and four of the study), recruitment of new participants was largely impeded. Data were often collected by phone, email, and text using contact information gathered during previous rounds of surveys. Third-year data were collected from May 2020 to December 2020, and fourth-year data from April 2021 to October 2021.

### 2.5 Data analysis

Missing data were common in this study because of the difficulties associated with longitudinal community-based research and the effects of the COVID-19 pandemic on participation rates. Specifically, 547 (46.7%) participants completed only one survey, 250 (21.3%) participants completed two surveys, 214 (18.3%) participants completed three surveys, and 160 (13.7%) participants completed a survey all four years. Linear regression analysis was performed to assess if the missingness mechanism impacted the aggregate outcomes for the human health, pet health, environmental health, community health, and perceived links subscales. A variable for the availability to complete a follow-up survey the year after the initial time-point of data collection was created to use as a proxy to assess missingness. Explanatory variables included in the regression model were survey date, education level, household income, sex, age, ethnicity, preferred language, and born in the U.S. Results revealed no significant relationship between the availability to follow up and the five aggregate outcome variables of interest in the study. These findings provide qualitative evidence that the data are missing at random, where the propensity for data to be missing is not inherent to the missing data but rather dependent on another variable (28). This evidence supports that the missingness mechanism did not create bias in the responses over time.

Generalized Estimating Equations (GEE) were used to analyze changes in overall perceived measures of One Health in phases one and two of the study and access to human healthcare, pet care, and the environment in phase one of the study. This statistical approach allowed longitudinal data analysis while considering multiple relevant covariates, even when the mathematical relationship between independent and dependent variables contained biased coefficients and parameter estimations. An exchangeable working correlation structure was chosen for these data and additional Wald tests were added to account for any misspecifications in the structure of assumed correlations (28–32). Analyses were conducted using SPSS version 25. As previously reported, propensity score matching was utilized to eliminate any demographic bias in the sample, resulting in a total of 512 urban participants and 234 rural participants (21). The following independent factors were included in each model as they could potentially affect the outcomes: preferred language, sex, age, race/ethnicity, household income, highest level of education completed, born in the U.S., survey date, and PFL intervention. The variables for preferred language were Spanish and “other,” with English being the reference category. Sex was measured as female and “other,” with male being the reference category. Age was measured in a range of years, including 18–30, 30–45, and 45–60 and a reference of 60 or older. The dichotomous variables for race/ethnicity included Black, Latino/a, and “other,” with White as the reference category. Household income was measured as $60,000 or more, $45,000– $60,000, $30,000–$45,000, $15,000–$30,000, with $0–15,000 as the reference category. Highest level of education was measured as college degree and high school degree or equivalent, with less than a high school degree as the reference category. Response options for the discrete variable, born in the U.S., were yes or no, with no serving as the reference category. For all demographic questions, “prefer not to answer” was provided as a response option. Survey date, included to help analyze changes over time, was measured as a continuous variable. For the GEE analyses in the second phase of the study, a binary variable for the intervention was used to define if PFL was present or absent in the community at the time of the survey. The intervention variable was calculated using PFL’s first contact with clients in the community and the survey date. If the survey was completed after the intervention was initiated in the community, the PFL intervention was “yes.” If the survey was completed before the intervention was initiated in the community, the PFL intervention variable was “no,” which served as the reference category. Interpretation of GEE results is based on the Likert scale in this study. Numbers represent the average change in Likert scale responses, either aggregated across domains and access subscales, or within a specific item, associated with the presence of PFL.

GEE analyses were run for the two-year dataset (phase one of the study) on the aggregated and disaggregated measures of access to the five OHCA subscales and the three subsets of items measuring perceptions of access. GEE analyses for the four-year data set (phase two of the study) were run on the five subscale measures of One Health at the end of the four-year period. Minor adjustments were made to the survey over the four-year longitudinal study for instrument validation purposes. Therefore, in the second phase analyses, 21 questions were removed from the analysis because they were not asked consistently across the study period. Only active responses to the OHCA were included in the analysis (i.e., prefer not to answer and not applicable responses were omitted). For the aggregate scores, the threshold for active responses was 10% to eliminate bias from missing data (33). Aggregated scores were created for each of the five subscales by averaging the survey responses in each subscale. Six items necessitated reverse scoring, and an additional seven questions were removed from the aggregate scores due to a high rate of nonresponse (over 10%). An additional individual factor was included in subsequent GEE analyses to account for the impact of COVID-19 on the confirmatory half of the second phase of the study. If a survey was completed after the official declaration of the COVID-19 pandemic on March 11, 2020 (34) the COVID-19 impact was counted as “yes.” If the survey was completed before the official declaration of the COVID-19 pandemic, the COVID-19 variable was “no,” which served as the reference category.

## 3 Results

Over the four-year study period, 2,329 OHCA surveys were completed with 1,171 participants (Table 1). Of the completed surveys, 722 (31%) were gathered in year one; 687 (29.5%) were gathered in year two; 393 (16.9%) were gathered in year three; and 527 (22.6%) were gathered in year four. Of the total, 2,066 (88.7%) were conducted in English and 263 (11.3%) surveys were conducted in Spanish. Survey completion numbers were 34.7% lower in the third and fourth year of the study when the COVID-19 pandemic limited in-person recruitment and survey collection.

**Table 1:** Demographics of participants in the sample (*N* = 1,171).

Variable	Study site	Total survey frequency across four-year study	Percent of total sample
Study site	Madison	371	31.7%
	Seattle	331	28.3%
	Granger	281	24.0%
	Wilder	188	16.0%
Preferred language	English	983	83.9%
	Spanish	177	15.1%
	Other	8	0.7%
	Prefer not to answer	3	0.3%
Sex	Male	403	34.4%
	Female	755	64.5%
	Other	5	0.4%
	Prefer not to answer	8	0.7%
Age	Older than 60 years old	241	20.6%
	45–60 years old	324	27.7%
	30–45 years old	344	29.4%
	18–30 years old	252	21.5%
	Prefer not to answer	10	0.8%
Ethnicity	White	590	50.4%
	Black	95	8.1%
	Latino/a	356	30.4%
	Other	119	10.2%
	Prefer not to answer	11	0.9%
Born in the U.S.	Yes	919	78.5%
	No	232	19.8%
	Prefer not to answer	20	1.7%
Household income	$0–$15,000	161	13.7%
	$15,000–$30,000	186	15.9%
	$30,000–$45,000	161	13.7%
	$45,000–$60,000	131	11.2%
	Greater than $60,000	274	23.4%
	Prefer not to answer	258	22.1%
Highest level of education	Less than a high school degree	199	17.0%
	At least a high school degree or equivalent	612	52.3%
	Bachelor’s degree or higher	330	28.2%
	Prefer not to answer	30	2.5%

### 3.1 Phase one: two-year findings

#### 3.1.1 Changes in overall perceptions of One Health subscales associated with PFL

The urban site that received the PFL intervention was associated with significantly increased perceptions of community health, by a magnitude of 0.109 (*p* = 0.006) and environmental health, by a magnitude of 0.130 (*p* < 0.001), than the urban site that did not have the PFL intervention. The measured perceptions of human health, pet health, and the perceived links between the One Health domains were not associated with an overall significant positive or negative difference between the intervention and the comparison community. A further breakdown of the results for the aggregate measure of the One Health subscales within the urban community by demographic level variables is presented in Table 2.

**Table 2:** Results of Generalized Estimating Equation analysis of how the presence of PFL in an urban community across two years, influences aggregated measures of community-wide health under the One Health framework.

Urban: two years	Community health	Human health	Pet health	Environmental health	Perceived links
Preferred language					
Spanish	0.150	−0.029	**−0.219 (−0.416, −0.023)**	−0.099	−0.076
Other	−0.045	−0.086	**−0.263 (−0.404, −0.122)**	−0.142	**−0.272 (−0.445, −0.099)**
Sex					
Female	−0.044	0.000	**0.055 (0.002, 0.108)**	**−0.068 (−0.134, −0.002)**	0.000
Other	−0.015	0.069	**0.385 (0.121, 0.648)**	0.097	0.361
Ethnicity					
Black	0.080	**0.107 (0.004, 0.209)**	−0.050	0.051	−0.046
Latino/a	0.001	−0.008	−0.072	0.015	−0.019
Other	0.009	0.017	−0.064	0.000	0.000
Age (years)					
45–60	−0.105	−0.062	−0.004	**−0.110 (−0.199, −0.020)**	−0.001
30–45	**−0.177 (−0.289, −0.065)**	−0.015	0.030	**−0.186 (−0.283, −0.088)**	−0.044
18–30	**−0.250 (−0.395, −0.105)**	−0.009	−0.020	**−0.239 (−0.349, −0.129)**	−0.015
Highest level of education completed					
High school degree or equivalent	0.006	−0.010	0.037	−0.042	−0.017
College degree	0.038	0.024	0.069	−0.052	0.014
Household income					
$15,000–$30,000	**0.194 (0.031, 0.357)**	0.037	−0.009	**0.16 (0.027, 0.293)**	−0.045
$30,000–$45,000	0.150	−0.038	−0.039	0.075	−0.053
$45,000–$60,000	0.108	0.060	−0.056	0.083	−0.090
>$60,000	**0.277 (0.131, 0.423)**	**0.197**	0.048	**0.244 (0.122, 0.366)**	−0.025
Born in the U.S.					
No	−0.063	−0.015	0.008	−0.017	−0.020
PFL intervention					
Yes	**0.109 (0.032, 0.185)**	0.037	0.017	**0.130 (0.065, 0.196)**	4.68 E-5
Survey date	**−0.004 (−0.069, 0.060)**	**−0.058 (−0.113, −0.004)**	**−0.075 (−0.129, −0.021)**	**−0.089 (−0.147, −0.032)**	**−0.041 (−0.093, −0.011)**

Negative numbers indicate decreased perceptions, and positive numbers indicate increased perceptions when analyzed against the comparison community.

All significant findings are bolded (*p* < 0.05), and 95% confidence intervals are reported in parenthesis for all significant findings. The reference categories are described in greater detail in the section “Generalized Estimating Equations.”

The rural site that received the PFL intervention was associated with significantly lower perceptions of human health, by a magnitude of −0.154 (*p* < 0.001), and perceived links between the One Health domains by a magnitude of −0.166 (*p* < 0.001). The measured perceptions of community, pet, and environmental health did not return a significant association with the PFL intervention. A further breakdown of the results for the aggregate measure of the One Health subscales within the rural community by demographic level variables is presented in Table 3.

**Table 3:** Results of Generalized Estimating Equation analysis of how the presence of PFL in a rural community across two years influences aggregated measures of community-wide health under the One Health framework.

Rural: two years	Community heath	Human health	Pet health	Environmental health	Perceived links
Preferred language					
Spanish	0.026	0.006	−0.071	−0.009	0.001
Sex					
Female	0.054	0.070	0.055	−0.007	0.019
Ethnicity					
Black	0.277	−0.154	0.006	**0.344 (0.085, 0.604)**	0.144
Latino/a	0.057	−0.002	−0.006	−0.015	−0.047
Other	−0.039	0.082	−0.138	0.072	−0.107
Age (years)					
45–60	−0.050	−0.020	−0.001	**−0.168 (−0.302, −0.034)**	−0.019
30–45	**−0.169 (−0.308, −0.029)**	−0.008	−0.021	**−0.149 (−0.269, −0.028)**	**−0.092 (−0.179, −0.004)**
18–30	−0.109	0.017	−0.004	−0.110	−0.025
Highest level of education completed					
High school degree or equivalent	0.126	**0.093 (0.002, 0.184)**	0.070	−0.063	0.051
College degree	**0.21 (0.022, 0.399)**	**0.17 (0.033, 0.307)**	**0.175 (0.039, 0.311)**	0.083	0.120
Household income					
$15,000–$30,000	0.169	**0.117 (0.005, 0.228)**	**0.147 (0.042, 0.252)**	**0.155 (0.012, 0.298)**	0.050
$30,000–$45,000	0.174	**0.165 (0.053, 0.277)**	**0.183 (0.062, 0.304)**	0.116	0.051
$45,000–$60,000	0.004	0.096	0.111	−0.017	−0.090
>$60,000	**0.222 (0.016, 0.428)**	**0.182 (0.041, 0.323)**	**0.274 (0.146, 0.402)**	0.061	0.065
Born in the U.S.					
No	0.002	**−0.113 (−0.220, −0.007)**	**−0.133 (−0.250, −0.015)**	0.003	0.007
PFL intervention					
Yes	−0.085	**−0.154 (−0.236, −0.072)**	−0.080	−0.089	**−0.166 (−0.252, −0.079)**
Survey date	**0.121 (0.057, 0.185)**	**0.118 (0.063, 0174)**	**0.137 (0.028, 0.193)**	**0.114 (0.051, 0.178)**	**0.137 (0.081, 0.193)**

Negative numbers indicate decreased perceptions, and positive numbers indicate increased perceptions, when analyzed against the comparison community.

All significant findings are bolded (*p* < 0.05), and 95% confidence intervals are reported in parenthesis for all significant findings. The reference categories are described in greater detail in the section “Generalized Estimating Equations.”

#### 3.1.2 Changes in overall perceptions of One Health access scales associated with PFL

Results of the GEE analysis for the aggregate measures of perceived access to pet care were previously reported in Hawes et al. (21). Overall, it was found that PFL was associated with increased perceptions of access to pet supportive services in the urban community (21).

The urban site that received the PFL intervention was associated with an increased aggregate measure of perceived access to human healthcare, by a magnitude of 0.163 (*p* < 0.001) compared to the urban site that did not have PFL present. In the rural community, the presence of PFL did not have a statistically significant association with the aggregate measure of perceived access to human healthcare. A further breakdown of the results for the aggregate measure of the access to human health scale within the urban and rural communities by the demographic level variables is presented in Table 4.

**Table 4:** Results of Generalized Estimating Equation analysis of how the presence of PFL in an urban and rural community influences aggregated measures of perceived access to human health and the environment over the two year study period (2017–2019).

	Urban: access to human health	Rural: access to human health	Urban: access to the environment	Rural: access to the environment
Preferred language				
Spanish	−0.097	−0.128	**−0.321 (−0.643, 001)**	−0.128
Other	0.102	0	**−0.782 (−1.457, −0.106)**	0
Sex				
Female	−0.015	−0.034	−0.062	0.025
Other	0.005	0	0.318	0
Ethnicity				
Black	0.106	−0.269	0.047	−0.54
Latino/a	−0.019	**0.246 (0.006, 0.487)**	−0.109	−0.042
Other	−0.067	0.101	0.058	0.192
Age (years)				
18–30	−0.002	−0.061	−0.075	−0.095
30–60	−0.002	0.06	−0.069	−0.126
Highest level of education completed				
High school degree or equivalent	**−0.142 (−0.267, −0.018)**	0.096	−0.054	−0.029
College degree	−0.111	0.083	0.05	0.135
Household income				
$15,000–$30,000	0.035	0.11	**0.200 (0.040, 0.359)**	**0.311 (0.066, 0.557)**
$30,000–$45,000	0.014	0.105	**0.197 (0.014, 0.379)**	0.119
$45,000–$60,000	−0.021	0.03	0.176	0.304
>$60,000	0.13	0.159	**0.260 (0.094, 0.426)**	**0.383 (0.113, 0.653)**
Born in the U.S.				
No	−0.013	**0.261 (0.100, 0.422)**	−0.08	0.025
PFL intervention				
Yes	**0.163 (0.078, 0.249)**	0.77	**0.120 (0.027, 0.214)**	−0.046
Survey date	**−0.104 (−0.183, −0.026)**	0.065	**−0.179 (−0.269, −0.088)**	0.051

Negative numbers indicate decreased perceptions, and positive numbers indicate increased perceptions, when analyzed against the comparison community.

All significant findings are bolded (*p* < 0.05), and 95% confidence intervals are reported in parenthesis for all significant findings. The reference categories are described in greater detail in the section “Generalized Estimating Equations.”

The urban site that received the PFL intervention was associated with an increased aggregate measure of perceived access to the environment, by a magnitude of 0.120 (*p* = 0.011), compared to the urban site without PFL. The presence of PFL in the rural community did not have a statistically significant association with the aggregate measure of perceived access to the environment. Table 4 presents a further breakdown of the results for the aggregate measure of the access to the environment scale within the urban and rural communities by demographic level variables.

#### 3.1.3 Changes in perceptions of the individual items within the One Health access scales associated with PFL

Results of the GEE analysis for the disaggregated measures of perceived access to pet care in the urban and rural communities receiving the PFL intervention were previously reported in Hawes et al. (21).

The urban community with the PFL intervention was associated with significantly greater perceived access to healthcare services that offer payment plans, by a magnitude of 0.191 (*p* = 0.018), and to human healthcare services in one’s geographic proximity, by a magnitude of 0.539 (*p* < 0.001), than the urban community without the PFL intervention. Significantly lower perceptions of access to human healthcare services in one’s preferred language were associated with the urban community receiving the PFL intervention by a magnitude of −0.122 (*p* = 0.005). Among the items included in the access to the environment scale, there were significantly greater perceptions of access to affordable options to enjoy the outdoors, by a magnitude of 0.193 (*p* < 0.001) associated with the urban intervention site. A further breakdown of the results for the disaggregated measures of the access to human health scale and access to the environment scale within the urban community by demographic level variables is presented in Table 5.

**Table 5:** Results of Generalized Estimating Equation analysis of how the presence of PFL in an urban community influences disaggregated measures of perceived access to human healthcare and access to the environment over the two year study period (2018–2019).

Urban	Human health: affordable options	Human health: affordable options (payment plans)	Human health: geographic proximity	Human health: preferred language	Human health: health information	Environmental health: affordable options	Environmental health: geographic proximity
Preferred language							
Spanish	0.029	−0.150	0.083	−0.253	−0.127	**−0.402 (−0.787, −0.017)**	−0.214
Other	**0.537 (0.101, 0.973)**	0.303	**0.685 (0.216, 1.154)**	**−0.569 (−0.841, −0.298)**	**−0.304 (−0.578, −0.030)**	−0.135	**−1.417 (−2.397, −0.437)**
Sex							
Female	−0.090	−0.052	0.037	0.055	−0.025	−0.032	−0.090
Other	0.205	−0.182	**−0.905 (−1.696, −0.115)**	**0.590 (0.092, 2.088)**	0.096	0.616	0.027
Ethnicity							
Black	**0.38 (0.182, 0.578)**	0.081	0.075	−0.049	0.093	0.026	0.066
Latino/a	0.083	0.217	−0.041	−0.156	−0.099	−0.089	−0.139
Other	−0.174	0.078	0.071	**−0.232 (−0.397, −0.066)**	0.006	0.020	0.079
Age (years)							
18–30	−0.026	0.135	−0.036	0.074	−0.048	−0.037	−0.111
30–60	−0.013	0.030		0.068	−0.002	−0.061	−0.081
Highest level of education completed							
High school degree or equivalent	−0.191	−0.216	−0.140	−0.004	**−0.147 (−0.290, −0.005)**	−0.101	0.002
College degree	−0.083	−0.303	**−0.250 (−0.466, −0.035)**	0.150	−0.097	−0.017	0.116
Household income							
$15,000–$30,000	−0.121	0.041	0.038	0.108	0.127	**0.287 (0.093, 0.481)**	0.131
$30,000–$45,000	**−0.372 (−0.714, −0.030)**	−0.036	0.208	0.086	0.091	0.137	**0.28 (0.096, 0.464)**
$45,000–$60,000	**−0.35 (−0.686, −0.013)**	−0.151	0.149	0.104	0.043	**0.291 (0.080, 0.503)**	0.082
>$60,000	0.002	0.149	0.153	**0.228 (0.087, 0.370)**	**0.199 (0.004, 0.394)**	**0.312 (0.110, 0.515)**	**0.224 (0.057, 0.392)**
Born in the U.S.							
No	0.120	−0.005	0.083	−0.049	−0.104	−0.057	−0.112
PFL intervention							
Yes	0.159	**0.191 (0.033, 0.349)**	**0.539 (0.390, 0.687)**	**−0.122 (−0.207, −0.037)**	0.066	**0.193 (0.087, 0.299)**	0.055
Survey date	−0.001	−0.094	**−0.230 (−0.364, −0.095)**	−0.024	**−0.156 (−0.252, −0.059)**	**−0.156 (−0.267, −0.045)**	**−0.161 (−0.269, −0.052)**

Negative numbers indicate decreased perceptions, and positive numbers indicate increased perceptions, when analyzed against the comparison community.

All significant findings are bolded (*p* < 0.05), and 95% confidence intervals are reported in parenthesis for all significant findings. The reference categories are described in greater detail in the section “Generalized Estimating Equations.”

Among the rural communities, the PFL intervention was associated with a significant increase in perceived access to healthcare services in one’s geographic proximity by a magnitude of 0.232 (*p* = 0.025). Neither individual item within the access to the environment scale returned a significant measure associated with the PFL intervention in the rural community. Table 6 presents a further breakdown of the results for the disaggregated measures of the access to human health items and access to the environment items in the rural community by demographic level variables.

**Table 6:** Results of Generalized Estimating Equation analysis of how the presence of PFL in a rural community influences disaggregated measures of perceived access to human healthcare and the environment over the two year study period (2017–2019).

Rural	Human health: affordable options	Human health: affordable options (payment plans)	Human health: geographic proximity	Human health: preferred language	Human health: health information	Environmental health: affordable options	Environmental health: georgraphic proximity
Preferred language							
Spanish	−0.105	−0.262	−0.090	−0.091	−0.121	−0.087	−0.157
Sex							
Female	0.143	−0.682	0.262	−0.050	−0.022	−0.020	0.053
Ethnicity							
Black	−0.493	0.243	−0.463	−0.025	−0.491	−0.402	−0.697
Latino/a	0.119	0.904	0.183	−0.017	**0.202 (0.043, 0.362)**	0.007	−0.080
Other	−0.164	0.018	0.183	0.152	**0.283 (0.038, 0.529)**	0.044	0.333
Age (years)							
18–30	0.133	−0.164	−0.097	−0.005	−0.129	0.112	**−0.311 (−0.522, −0.100)**
30–60	0.026	0.356	0.011	0.085	−0.045	−0.051	−0.189
Highest level of education completed							
High school degree or equivalent	0.077	0.144	0.066	0.136	0.058	−0.085	0.048
College degree	0.273	−0.808	0.291	**0.290 (0.020, 0.560)**	0.105	0.083	0.204
Household income							
$15,000–$30,000	0.074	−0.174	0.275	0.027	0.164	0.300	**0.348 (0.071, 0.625)**
$30,000–$45,000	0.056	−0.124	0.179	0.013	0.229	0.172	0.072
$45,000–$60,000	−0.072	−0.145	0.040	0.028	**0.261 (0.020, 0.502)**	0.288	0.308
>$60,000	−0.111	1.032	−0.074	0.068	0.086	**0.494 (0.139, 0.849)**	0.269
Born in the U.S.							
No	0.202	0.493	0.137	**0.242 (0.068, 0.416)**	**0.283 (0.145, 0.421)**	0.034	−0.019
PFL intervention							
Yes	−0.043	0.392	**0.232 (0.029, 0.434)**	−0.005	−0.061	−0.035	−0.067
Survey date	0.007	0.342	0.04	0.035	−0.003	0.054	0.042

Negative numbers indicate decreased perceptions, and positive numbers indicate increased perceptions, when analyzed against the comparison community.

All significant findings are bolded (*p* < 0.05), and 95% confidence intervals are reported in parenthesis for all significant findings. The reference categories are described in greater detail in the section “Generalized Estimating Equations.”

### 3.2 Phase two: four-year findings

#### 3.2.1 Changes in overall perceptions of One Health subscales associated with PFL

PFL presence in the communities was associated with a higher aggregate measure of environmental health at the four-year mark, by a magnitude of 0.048 (*p* = 0.033), compared to when the PFL intervention was not present in the communities. Table 7 presents a further breakdown of the results for the aggregate measure of the One Health subscales at the four-year measure associated with the presence of PFL in a community by demographic level variables.

**Table 7:** Results of Generalized Estimating Equation analysis of how the presence of PFL across all four communities over four years influences aggregated measures of community-wide health under the One Health framework.

	Community heath	Human health	Pet health	Environmental health	Perceived links
Preferred language					
Spanish	0.049	0.004	**−0.089 (−0.174, −0.005)**	−0.016	−0.027
Other	−0.280	0.004	**−0.170 (−0.281, −0.059)**	−0.374	**−0.276 (−0.479, −0.073)**
Sex					
Female	−0.014	0.038	**0.058 (0.018, 0.097)**	−0.046	0.015
Other	−0.065	−0.294	0.165	0.049	0.239
Ethnicity					
Black	0.084	0.072	−0.013	**0.099 (0.022, 0.177)**	0.014
Latino/a	0.087	0.039	−0.049	−0.049	**−0.139 (−0.201, −0.077)**
Other	−0.065	0.030	−0.048	−0.004	−0.036
Age (years)					
45–60	−0.082	**−0.065 (−0.126, −0.005)**	−0.007	**−0.095 (−0.167, −0.023)**	0.007
30–45	**−0.180 (−0.262, −0.098)**	−0.035	−0.013	**−0.165 (−0.236, −0.095)**	**−0.070 (−0.121, −0.019)**
18–30	**−0.154 (−0.247, −0.061)**	0.003	0.012	**−0.162 (−0.238, −0.086)**	0.002
Highest level of education completed					
High school degree or equivalent	0.033	0.050	**0.064 (0.009, 0.118)**	**−0.074 (−0.142, −0.006)**	0.048
College degree	0.051	0.057	**0.105 (0.037, 0.174)**	−0.013	**0.125 (0.055, 0.194)**
Household income					
$15,000–$30,000	**0.227 (0.112, 0.341)**	**0.092 (0.017, 0.167)**	0.070	**0.211 (0.121, 0.302)**	0.054
$30,000–$45,000	**0.263 (0.150, 0.376)**	**0.083 (0.005, 0.161)**	**0.076 (0.000, 0.152)**	**0.200 (0.108, 0.292)**	0.039
$45,000–$60,000	**0.154 (0.020, 0.288)**	**0.099 (0.018, 0.181)**	0.036	**0.139 (0.033, 0.245)**	−0.007
>$60,000	**0.343 (0.230, 0.457)**	**0.215 (0.137, 0.293)**	**0.168 (0.095, 0.241)**	**0.261 (0.169, 0.353)**	**0.084 (0.015, 0.154)**
Born in the U.S.					
No	−0.016	**−0.087 (−0.155, −0.020)**	−0.062	0.022	0.040
PFL intervention					
Yes	0.032	0.003	0.019	**0.048 (0.004, 0.092)**	0.004
Survey date	−0.018	0.012	**0.019 (0.002, 0.036)**	**−0.021 (−0.040, −0.001)**	**0.020 (0.005, 0.036)**

Negative numbers indicate decreased perceptions, and positive numbers indicate increased perceptions.

All significant findings are bolded (*p* < 0.05), and 95% confidence intervals are reported in parenthesis for all significant findings. The reference categories are described in greater detail in the section “Generalized Estimating Equations.”

The COVID-19 pandemic, analyzed in subsequent GEE models as an additional factor, was found to have no significant impact on surveys taken before and after the declaration of the COVID-19 pandemic for four of the five subscales. Among the human health subscale, a significant increase, by a small magnitude of 0.061 (*p* = 0.016), was observed in perceptions of human health among surveys taken during the pandemic with the PFL intervention present. Due to the unforeseen circumstances of a global pandemic and the predetermined study design laying out all four sites to be intervention sites starting in April of 2020, a comparison of communities having received the PFL intervention and communities having not received the PFL intervention during the pandemic years is not able to be presented. Because there was no comparison site utilized where one could understand the associated impacts of COVID-19 alone on individuals’ perceptions of One Health, results including the COVID-19 pandemic variable are not reported in this manuscript.

## 4 Discussion

With more than 1,100 individuals participating over four years, many over multiple annual data collection points, the study reported here aims to understand community-level perceptions of the individual domains and potential interconnections within the One Health framework. The PFL intervention infused resources into the companion animal domain of the triad, representing a less anthropocentric approach to addressing One Health issues. Comparison groups were created by utilizing publicly available data to identify urban and rural communities with similar demographic profiles and degrees of resource availability, and an innovative and validated instrument, the OHCA, was then used to longitudinally assess individual residents’ perspectives of the One Health domains. The first two years of this study allowed for the observation of the five OHCA subscales and perceived access to components of One Health between urban and rural pairs, with one of each receiving the intervention. The second two-year study period, which sought to confirm changes observed in the first phase of the study and holistically assess perceptions of One Health when all four communities were receiving the intervention, was impacted by the onset of the COVID-19 pandemic. Intervention implementation and data collection were substantially hindered, and the necessitated public health measures likely effected individuals’ perspectives of components of One Health. Therefore, the results prompt consideration within both One Health and public health contexts.

### 4.1 Phase one: two-year results

In the assessment of the five One Health subscales during the first two years of the study, a significant increase in perceptions of community health was observed in the urban site receiving the PFL intervention. When resources are focused on the companion animal domain of the One Health triad within urban settings, the overall perceived health of the community may be fostered. This is consistent with previous research on the effects of increasing social support to address SDH (35, 36). For example, a scoping review by Nickel and von dem Knesebeck (37) confirmed that a majority of community-based health promotion and prevention programs in urban environments result in at least one reported improvement in health status or behavior and that this is particularly evident when the intervention is focused on building the resources and capacity of the community in focus. Feeling invested in one’s community, and that one’s community is equally invested in them, has a considerable impact on people’s health and well-being. Michalski et al. (35) found that individuals experiencing a very strong sense of community belonging were associated with significantly increased odds of reporting better physical and mental health compared to those identifying as having a very weak sense of community belonging. This effect may extend to investment in addressing SDAH for a community’s pets, which directly affects family cohesion and well-being (16).

The first phase, two-year findings also support that a pet-focused intervention may increase an urban community’s perception of environmental health. Similar to an individual’s understanding of community health, one’s perception of their built and natural environment lends itself relevant to physical and mental health contexts (38). Significantly lower rates of psychological distress (e.g., depression, anxiety, stress), accompanied by improvements in mood, attention, and rates of physical activity, have been linked to increased time spent in nature, including urban nature settings (38, 39). Moreover, the acknowledgment and experience of natural spaces in one’s community have been associated with increased social mobility, more cohesive communities, and resilience in the face of public health crises, while physical increases in the amount of green space have been associated with decreases in levels of environmental pollutants and hazards (39).

While assessments of the other OHCA subscales did not return a significant aggregate difference associated with the PFL intervention, across both urban communities, regardless of the intervention, there were significant, increased perceptions of human health and pet health observed for individuals who identified as Black and with a gender other than male, when compared to white and male-identifying participants, respectively. These results indicate that among the urban communities under observation, there may be generally more positive understandings of human and pet health among populations that are systemically disadvantaged because of their race and/or gender. This could be due to a variety of local-level factors and dynamics.

The same One Health subscale assessment in the rural community indicated significantly decreased perceptions regarding human health and perceived links between the One Health domains, with the other subscales trending downward. Conceptualizations of health differ greatly along the rural–urban continuum due to factors related to the economy, inequities regarding one’s race and ethnicity, population density, resource distribution, and drastic shifts in the spatial concentration of poverty (40). The rural community findings, in antithesis to those observed in the urban community, suggest that there are complicated dynamics present in the rural area that are beyond the scope of the intervention design. This is particularly likely given that the intervention reached a higher percentage of households in the urban areas and that the OHCA was initially informed by a focus group from an urban community (20). A better understanding of the sociological interplay of rural communities could help inform community-specific strategies and interventions that would more appropriately address both SDH and SDAH in rural areas (41).

Fourteen OHCA access items were also evaluated across the first two years of the study. As previously reported, a significant increase in perceptions of access to veterinary care was observed in the urban site receiving the PFL intervention (21). Here, we report that these findings in the urban intervention community extend to perceptions of access regarding human health care and the environment.

#### 4.1.1 Access to human health

In this study, perceived access to human health care services significantly increased in the urban community receiving the PFL intervention. Therefore, the infusion of resources into the companion animal component of the One Health triad may positively impact an individual’s understanding of their access to human health services. The disaggregated analysis of the access to human health scale identified a significant increase in the perception of access to payment plan options in human healthcare settings in urban communities receiving the PFL intervention. Like the accessibility of pet support services, affordability is one of the most often cited barriers to accessing human healthcare services (42, 43). While PFL was not associated overall with higher perceived access to affordable human healthcare services in either community receiving PFL services, the increased perception of access to payment plan options suggests that urban community members may feel that healthcare services are more affordable when a cost is able to be spread out over time. This could be attributed to PFL’s efforts to work with local animal welfare organizations to provide payment plan options for services provided. Moreover, the demographic breakdowns indicate that across both urban communities, intervention aside, there was a significantly higher perception of access to affordable healthcare services, for individuals identifying as Black and those speaking a language that is not English or Spanish, compared to White and English-speaking participants, respectively. In economically marginalized communities, lower perceived affordability of care persists across race, ethnicity, and language, and intersectionality analyses have also illustrated decreased perceived and actual differences in health for low-socioeconomic status (SES) Black individuals compared to low-SES White individuals (44–47). The increased perceptions seen among the specific demographic groups here indicate that a variety of factors at play, including PFL, could be influencing the results.

The disaggregated analysis also indicated an increased perception of access to human healthcare services within one’s geographic proximity for both the urban and rural communities that had the PFL intervention present. Geographic access is one of several factors influencing one’s overall access to care, and, perhaps more importantly, research has shown that increased geographic access to human healthcare services is associated with a greater utilization of those resources and overall improved health outcomes (48). These data do not quantitatively show that geographic access actually increased, however, the communities’ increased perceptions of geographic access could lead to higher healthcare utilization.

Access to healthcare services in one’s preferred language was the one disaggregated analysis that returned an overall significant decrease in perceptions associated with PFL in the urban community, mirroring the results regarding access to pet care in one’s preferred language (21). Hawes et al. (21) mention that this could be a result of the differing rates of diversity between the intervention site (Madison, WI) and the comparison site (Seattle, WA), which had a higher percentage of non-English speaking neighborhoods. Building language capacity is crucial within the human healthcare setting, as it has been identified that among specific racial and ethnic groups, there is a significantly decreased health status for those individuals who do not speak English (49).

#### 4.1.2 Access to the environment

A significant increase was observed in perceived access to the environment in the urban community receiving the intervention during the first two years of this study. Therefore, an infusion of resources into the companion animal component of the One Health triad could encourage a more positive realization of one’s access to natural spaces. Mirroring discussions around access to human healthcare and pet support services, affordability is one of the most often cited barriers when it comes to accessing natural spaces. When examining access to the outdoors, affordability factors heavily for historically marginalized and under-resourced communities, particularly when observing the price of gear needed for safe engagement in activities, entrance fees to national or state parks, transportation costs to access safe outdoor spaces, and unpaid time off from work (50, 51). PFL was associated with a significant increase in the perception of affordable options for enjoying the outdoors in the urban community in this study. PFL focuses heavily on providing and building out affordable pet care services within the communities. In realizing the resources available for accessing affordable veterinary care, individuals could have been encouraged to seek out additional support in their community for accessing affordable options for outdoor recreation, which could explain these findings.

In this study, there was no significant increase in perceived access to outdoor recreation within one’s geographic proximity for either the urban or rural communities receiving the PFL intervention. Green spaces and parks are often inequitably distributed (51). It has been documented that the quality and size of parks are significantly greater in high-SES, white neighborhoods, with low-SES and non-White communities often opting to travel outside of their neighborhood to larger parks with more amenities and higher safety ratings (51, 52). In rural communities specifically, it has been demonstrated that park proximity decreases as a community becomes more rural, with an average of only 7.8% of rural populations living within a half mile of a park (53). Therefore, historically, socially, and economically marginalized communities, like those under observation in this study, may be contiguous to parks. However, those parks are likely under-resourced, lack adequate amenities and maintenance, have a lower degree of safety, or require additional transportation to access, which are all things that PFL programming has no direct impact on in their service provisioning.

Overall, the results from the two-year analysis indicate that addressing structural barriers to accessing pet support services with the intentional deployment of veterinary resources into an urban community may measurably improve communities’ perceived access to pet care services, human health services, and the environment. Because there were few significant impacts of PFL found on rural community perceptions of access, further research should be conducted into the social ecology of rural communities and the additional supports such communities may require to gain access to One Health factors. Research has indicated that increased perceptions of access to health-promoting services can result in higher usage of those services (54). Thus, future research in this area should seek to understand if the intentional deployment of veterinary resources also quantitatively improves the utilization of pet support services, human health services, the environment, and active participation in one’s community.

### 4.2 Phase two: four-year results

While the results of analyses after the first two years of the study period provide some evidence supporting the hypothesis that the infusion of resources into communities to support pet health affects perceptions of both community health, environmental health, and access to the One Health triad, interpretation of the analysis of data from the entire four years of the study is complicated by the COVID-19 pandemic that bisected the study period. In the two sites receiving the PFL intervention from the outset, ongoing accessibility of services was hampered by community-wide lockdowns and additional restrictions on services to comply with emergency public health orders, while both the initiation and implementation of the PFL interventions were substantially hindered in the two comparison communities. This led to limited community knowledge of, and engagement with PFL services across the four study communities following the onset of the pandemic. Therefore, the community-based intervention under study during the latter half of the four-year period had a substantially lower rate of services than a typical PFL program. Further, data collection after the onset of the COVID-19 pandemic could not be conducted following the door-to-door method utilized during the first two years of the study because of public health precautions. Surveys were instead administered via existing phone and email contact information, resulting in a lower number of completed surveys in the confirmatory half of the study period. It is also likely that surveys completed by phone, or the internet performed differently than those surveys administered via in-person dialogue. Collectively, these limitations affected both the dosage (number of pet services per period of time) of the intervention during the second two years and the statistical power of the data.

Additionally, the data collected via the OHCA tool, designed to holistically measure perceptions of One Health, were likely substantially confounded by the COVID-19 pandemic, the most severe global One Health crisis to arise in the twenty-first century (55). No community was spared from the array of compounding social, physical, and economic impacts that resulted (56). Thus, within the context of COVID-19, negative perceptions of human, pet, and community health would be expected. However, this is particularly relevant for socially and economically marginalized communities, like those under observation in this study, which were disproportionately impacted by the cascade of pandemic effects (57). A breadth of data indicate that the pandemic caused severely elevated psychological effects, heightened states of poverty and segregation, detrimental disruptions in educational systems, and an extensive decline in interpersonal relationships and social capital, most notably in under-resourced neighborhoods (57). In this study, a subsequent GEE analysis was run to identify any distinctions in surveys taken before and during the pandemic associated with the PFL intervention, but the analysis indicated no significant differences, except for a fractional positive change in the human health subscale. It was not possible to analyze the isolated effect of COVID-19 on communities’ perceptions of the One Health subscales because no comparison site was utilized across all four years in which one could assess only the impacts of the pandemic on a community’s perceptions without the presence of PFL, and vice versa. Therefore, it is possible that PFL positively impacted One Health perceptions in the communities receiving their services across the four-year period, but this impact was overwhelmed by the COVID-19 pandemic’s effects. Therefore, due to the severity of One Health implications introduced by COVID-19, we interpreted the four-year study findings from the perspective of the pandemic, as opposed to the PFL intervention, being the largest driver behind perceptions measured by the OHCA.

With COVID-19 having contributed to an excess mortality of almost 15 million people globally, and over 700,000 deaths in the U.S. alone, between January 2020 and December 2021, resulting in mass lockdowns and other public health mandates, it is reasonable to assume that individuals understanding of human health security and their communities would be negatively impacted (58). However, mortality resulting from COVID-19 was not evenly distributed across communities, instead becoming more acute with increasing SDH risk factors. For example, reports found that COVID-19 infectivity and mortality were approximately 1.7 times greater for residents of the most economically marginalized U.S. counties, and for predominately Black counties, that rate jumped to between three and six times higher when compared to predominately White counties (57, 59). Brakefield and Authors (59) detail the additional pandemic challenges experienced within high-risk SDH communities during the pandemic. Residents of these communities often work low-wage, service jobs in essential work settings (e.g., grocery stores, transportation, cleaning services), consequently increasing susceptibility to COVID-19 infectivity and mortality. Moreover, the pandemic also brought with it unprecedented levels of unemployment, peaking in the United States at 14.7% in April of 2020; a statistic with a majority makeup of racial and ethnic minorities and those living in already economically vulnerable areas (60–62). Upticks in unemployment rates are typically correlated with increased food and housing insecurity, and those who lost their job during the pandemic faced “grim prospects of finding employment and losing health insurance” (60, 61). Studies also confirmed adverse psychological outcomes, such as increased reporting of anxiety, fear, and depression, with generally poor perceptions of health, in response to the COVID-19 pandemic and lockdowns (63–66). One report specifically found that among American adults, anxiety and depression were three times greater in 2020 than in 2019, with great prevalence among women, children, and older adults (57). Mental distress, such as anxiety and depression, can skew one’s perceived reality and intensify an already fragile mental state (67). Social relationships were also disrupted and declined dramatically due to the social isolation normed during the pandemic. Restrictions on social relationships during the pandemic were associated with increased stress, loneliness, domestic violence, and decreased feelings of friendship and belonging (57, 68). There is ample data showing the vast and detrimental impact that COVID-19 had on communities’ physical, mental, social, and economic health. These different health factors are all primary SDH informing one’s state of health and, therefore, the perception of a variety of health measures, such as those captured by the OHCA (15, 20). While not the intended measure of this study, it is likely that the global pandemic, a One Health crisis resulting in severe impacts on the social, economic, and physical and mental health of human communities, also negatively impacted how community and human health was perceived by the communities under observation in this study.

Similar to the impact of SDHs on human health from the onset of the pandemic, it can be inferred that SDAHs had a similar impact on pet health due to the effects of reoccurring lockdowns (16). Interviews conducted with individuals in a low-income community in Canada found that the pandemic elicited new barriers to accessing veterinary care that were associated with one’s socioeconomic status. For example, emergency veterinary appointments had to be accessed in lieu of one’s regular provider due to limited appointment availability, limited transportation options to and from appointments, communication difficulties with veterinarians that arose from telemedicine appointments, the need to comply with public health ordinances (e.g., masking, social distancing), and the ability to cover costs that were compounded by financial uncertainty during the pandemic. Collectively, these barriers resulted in increased stress and fear of losing one’s pet (69). A scoping review on the well-being of pets and their caregivers during COVID-19 across a spectrum of communities unearthed mixed results regarding the impacts on people’s relationships with their pets (70). Individuals found positive psychosocial benefits from being with their pets during lockdowns and working from home, physical and social benefits from increased exercise (e.g., dog owners), and many found that their relationship with their pet(s) improved (70). However, others reported challenges in not only being able to access the medical care and supplies needed for their pets but also in meeting their pet’s behavioral and social needs. Concerns and fear over what would happen to one’s pets if they were to become ill or hospitalized with COVID-19 were also expressed, with some individuals reporting that they would even delay testing or treatment for COVID-19 over such concerns (70). Within the reported study, PFL services from the onset of the pandemic had to be dramatically scaled back to comply with emergency public health regulations. Therefore, while pet owners in the communities under study likely had greater access to veterinary services during the pandemic, that access was also then impeded by new barriers introduced by the pandemic. This new reality for the landscape of pet care in the community, in addition to the disparity in impacts the COVID-19 pandemic elicited upon under-resourced and economically vulnerable communities, and likely pet health as well, probably had a negative impact on individual’s perceptions of their pet’s health overall.

While the human, community, and pet health domains of the OHCA did not change significantly over the four-year study period, a positive change was measured in the environmental health domain. While possibly affected by the concentrated infusion of pet support resources through PFL, this measure was more likely impacted by the COVID-19 pandemic and lockdowns that created a global slowing of human activity, recently coined the “anthropause” (71). Studies measuring worldwide environmental impacts during the height of the pandemic (January 1, 2020, to February 1, 2021), found that air quality improved, water ecosystems exhibited signs of recovery, wildlife sightings increased in urban areas, and there was a general decline in levels of human-generated pollution (i.e., air, water, soil, noise) (72). While there were also several negative environmental impacts during this time (e.g., increased medical waste, higher use of disinfectants, disruptions in ecosystems typically occupied by humans) (71, 72), media outlets around the world emphasized the positive; a stark contrast to the regular environmental programming on the triple planetary crisis, detailing the issues of climate change, biodiversity loss, and pollution (73). People were not just witnessing these impacts through the media, but they themselves were going outside more where they could observe changes in their environment. A recent study suggests that in the United States, approximately half of adults were outdoor recreation participants during the pandemic, with 20% identifying as new to the activity (74). Another survey study done in the United Kingdom found that the time people spent connecting with family and friends outdoors grew from 11 to 22% during the lockdown, and 36–46% of respondents indicated they were spending more time outside during the pandemic than they were before (74, 75). Due to the enormity of the COVID-19 pandemic and its grievous implications for One Health worldwide, it cannot be determined if the aggregate four-year measures are a result of the PFL intervention or COVID-19, though it is likely an unequal combination of the two. Future research will need to be done to confirm the impacts of PFL in a community over a four-year period isolated from major confounding variables.

## 5 Conclusion

The findings of the first two years of the study provide initial support that companion animal-focused interventions like PFL may significantly increase perceptions of the community health and environmental health OHCA subscales and the aggregate access items across all three components of the One Health triad, at least in urban communities. However, PFL seemed to be associated with decreased perceptions of these same OHCA measures found in the rural study sites, therefore, it cannot be ruled out that the pet-focused intervention may contribute to negative perceptions of the OHCA subscales. Across the entire four-year study period, perceptions of the environmental health domain of the OHCA showed positive changes that were associated with the presence of both the PFL intervention and COVID-19 pandemic public health mandates. This study offers insight into the impacts that the COVID-19 pandemic had on perceptions of One Health in the communities under observation that are consistent with previous research and identifies potential intervention points for mitigating the impacts that future pandemics may assert on individuals and their pets in under-resourced areas.

## 6 Limitations

In addition to the challenges introduced by the COVID-19 pandemic described above, several other factors may have affected the findings of the study. The design of the first phase required the comparison of two urban and two rural communities that had similar but not exactly matching demographics. An in-depth understanding of community-level dynamics within the zip codes under observation in this study could not be captured through census-level information alone. Therefore, it is possible that the observed correlations between PFL and higher perceived access to One Health could be due to other differences in the community that may not be generalizable, such as demographic factors or pet ownership rates, and other entities providing veterinary or pet supportive services (25). Moreover, the OHCA instrument, used across both the urban and rural communities in this study, was only informed through a pilot study completed in an urban community in Denver, Colorado (20). Urban and rural ecosystems differ drastically in terms of density, degree of built environment, resource availability, and social mobility. These factors inform an individual’s reality and, consequently, their perceptions. Therefore, the OHCA instrument may not have been the most appropriate way to assess the perceptions of individuals living in rural communities. One sees the difference in perception between these communities captured in the first phase analyses, where the PFL intervention had a significant positive association with the OHCA access scales only in the urban community and not the rural one. Future research should explore the development of an instrument like the OHCA as it pertains specifically to rural communities. Additionally, the second phase of the study aggregated the data collected across the urban and rural communities. It is possible that the aggregated urban and rural data in the second phase analysis do not holistically represent either the urban or rural communities, and future investigations should explore data from rural and urban areas separately.

Complex dynamics within communities beyond supportive interventions and public health crises can affect perceptions across the One Health framework. For example, the urban community in Seattle, WA was found to be experiencing gentrification during the second phase of this study. This poses a potential limitation to the study because gentrification has been found to be associated both positively and negatively with measures of community-wide health measured by the OHCA (76).

Finally, the OHCA instrument utilized in this study consisted of over one hundred questions aimed at assessing aspects of One Health. Long survey instruments, like the OHCA, are typically associated with lower response and completion rates and can introduce fatigue as a factor (77). When fatigue occurs, respondents can lose interest, resulting in the possibility of inaccurate answers or a high rate of non-response. Future research should explore the use of a shorter version of the OHCA to reduce complications associated with long survey instruments (20).

## Data Availability

The raw data supporting the conclusions of this article will be made available by the authors, without undue reservation.
